# Lemierre’s Syndrome: An Atypical Case of Fusobacterium necrophorum Bacteraemia in the Absence of Internal Jugular Vein Thrombosis

**DOI:** 10.7759/cureus.66867

**Published:** 2024-08-14

**Authors:** Astly George, Joel Thomas, Samanthi Welikumbura, Yash Achhapalia

**Affiliations:** 1 Department of Internal Medicine, Betsi Cadwaladr University Health Board, Wrexham, GBR; 2 Department of Microbiology, Betsi Cadwaladr University Health Board, Wrexham, GBR; 3 Department of Radiology, Betsi Cadwaladr University Health Board, Wrexham, GBR

**Keywords:** venous thrombosis (ijv), pharyngitis, pneumonia, cavitation, multiple pulmonary nodules, pleural effusion, malignancy, septic emboli, fusobacterium necrophorum, lemirre’s syndrome

## Abstract

Lemierre's syndrome primarily affects healthy adolescents and young adults as a complication of oropharyngeal infection, most commonly pharyngitis or peritonsillar abscess. *Fusobacterium necrophorum* is the principal pathogen, and the infection presents with classic symptoms including fever, sore throat, and neck tenderness. However, atypical presentations can pose diagnostic challenges. This report discusses a patient in her early 60s, contrary to the typical demographic, who presented with a one-week history of varied symptoms including sore throat, pleuritic chest pain, and haemoptysis. Examination revealed mild neck tenderness and lung crepitations. Laboratory tests indicated leucocytosis, thrombocytopenia, and elevated C-reactive protein (CRP). Imaging revealed pulmonary infiltrates with cavitation. *F. necrophorum* was detected in blood culture, promoting a CT scan of the neck, which confirmed soft tissue swelling and a small peritonsillar collection, leading to the diagnosis of Lemierre's syndrome. The classical feature of jugular vein thrombus was absent, further underscoring the atypical nature of this case. The patient received immediate initiation of intravenous antibiotics, piperacillin/tazobactam, followed by meropenem. This was complemented by a carefully tailored 21-day intravenous course, followed by an eight-week regimen of oral antibiotics consisting of amoxicillin and metronidazole. The patient demonstrated significant clinical improvement in pulmonary complications. Follow-up imaging showed minor residual changes, and the patient remained asymptomatic. Lemierre's syndrome presents a diagnostic challenge due to diverse clinical manifestations. Key diagnostic markers include deep neck infections, septicemia, and metastatic infections. Timely utilization of diagnostic tools, such as blood cultures and imaging, aid in confirmation. Early diagnosis is crucial for prompt treatment and prevention of complications. This case emphasizes the importance of maintaining a high index of suspicion for Lemierre's syndrome, especially in atypical presentations. Increased awareness among healthcare providers is vital for timely diagnosis and optimal patient outcomes.

## Introduction

Lemierre's syndrome was initially described by a French physician, André Lemierre, who reported 20 cases in 1936 of which only two survived. Lemierre's syndrome primarily affects healthy adolescents and young adults. It typically arises as a complication of oropharyngeal infection, most commonly pharyngitis or peritonsillar abscess [1]. Although this illness has been infrequent in the antibiotic era, it has been reported more in the past decade, probably due to rising resistance to antibiotics or reduced antibiotic prescriptions for upper respiratory tract infections [2].

An anaerobic bacterium, *Fusobacterium necrophorum*, is the principal pathogen associated with this syndrome. While classical presentations include fever, sore throat, and neck tenderness, atypical cases may pose diagnostic challenges, necessitating a comprehensive clinical assessment and heightened vigilance from clinicians [1,3]. We present here an atypical case of Lemierre's syndrome where the patient presented with respiratory symptoms. Similar cases with varied presentations have been reported in the literature, including cases with cutaneous manifestations such as cellulitis or abscesses [1], and cases with neurological symptoms due to septic emboli or meningitis [3].

## Case presentation

A female patient in her 60s presented with a two-week history of diverse clinical manifestations, including continuous fever with rigours, sore throat, cough with occasional mild haemoptysis, central stabbing chest pain, headache, nausea, vomiting, diarrhoea, fatigue, and lower back pain. There was no history of rash, lumps, unintentional weight loss, recent travel or surgical procedures. The patient had a medical history significant for prediabetes and non-toxic nodular goitre and came from a family with a strong history of diabetes, ischaemic heart disease and hypertension.

The patient's vital signs revealed a high-grade fever of 40 degrees Celsius, tachycardia at 110 beats per minute, normotension at 110/62 mmHg, and hypoxia with an oxygen saturation of 88% on room air. On physical examination, she was pale and tender over the right sternocleidomastoid, with a palpable small solid nodule on the left thyroid lobe. Additionally, oral examination revealed a black hairy tongue and slight asymmetry of the anterior pillars, which were thicker on the left side. There were no signs of icterus, clubbing, lymphadenopathy, or peripheral oedema. On auscultation, crepitations were noted more towards the base bilaterally, with normal heart sounds. Other systemic examinations were unremarkable.

Laboratory investigations revealed neutrophilic leucocytosis, thrombocytopenia, elevated inflammatory markers, and deranged liver function tests as shown in Table 1.

**Table 1:** Laboratory investigations on admission

Parameter	Result	Normal range
White blood cells	26.4 x 10^9^	4-11 x 10^9^
Platelet	15 x10^9^	150-400 x 10^9^
Hemoglobin	87	115-165 g/L
Neutrophils	24.1	1.7-7.5 x 10^9^
C-reactive protein	247	<3 mg/L
Bilirubin	25	<21 µmol/L
Alkaline phosphatase	183	30-130 U/L
Alanine transaminase	38	<33 U/L
Albumin	14	35-50 g/L
Prothrombin time	11.5	9-12 sec
Activated partial thromboplastin time	24.2	23-33 sec
Fibrinogen	8.1	2-4 g/L
d Dimer	1232	<500 ng/mL

Given the complexity of her presentation, non-specific findings, leucocytosis with neutrophilia and deranged liver function on admission, the patient was started on piperacillin-tazobactam 4.5 gm four times a day, as per the hospital guidelines for infection of unknown source.

**Figure 1:**
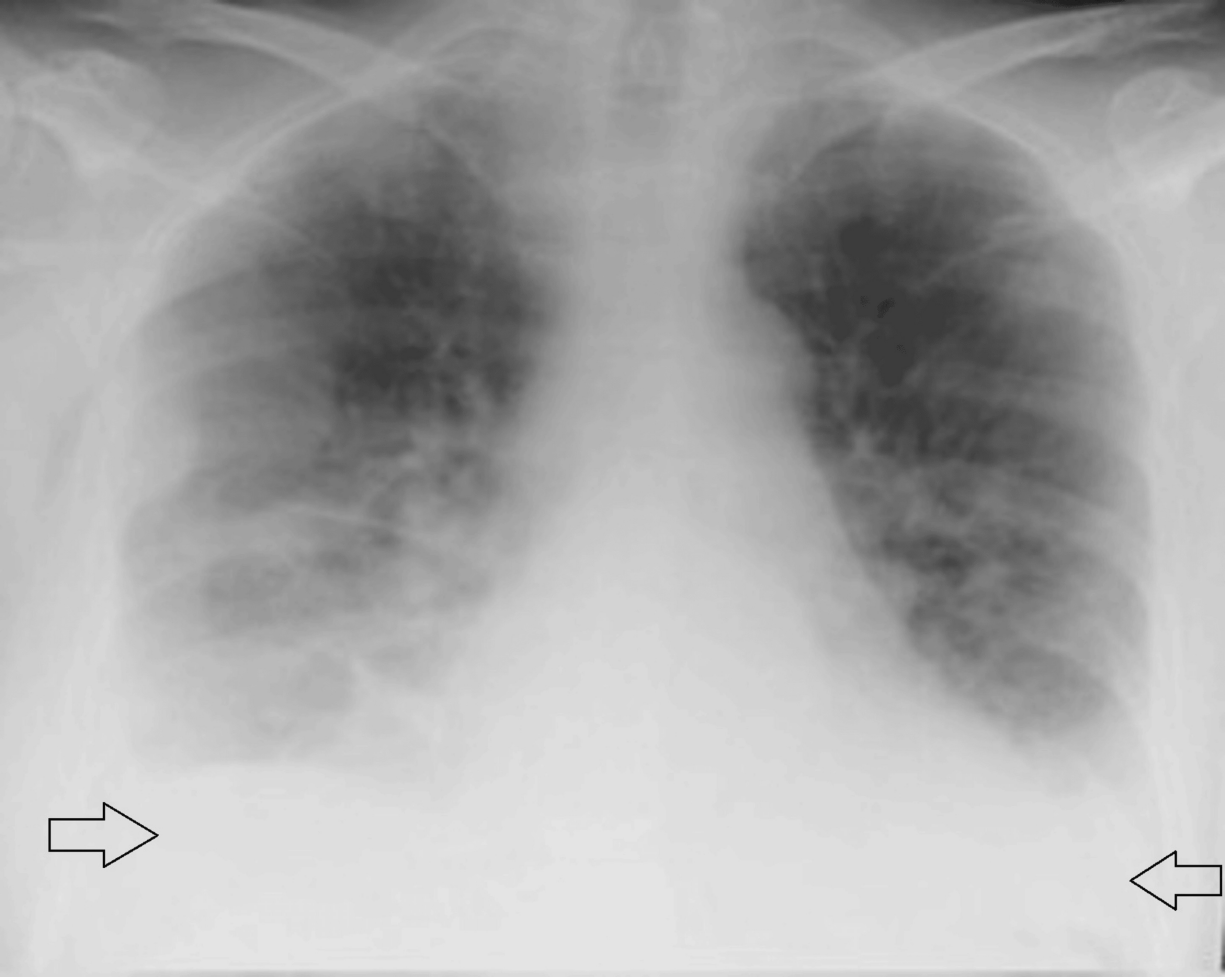
Chest X-ray showing significant bi-basal consolidation and small bilateral effusions (black arrows).

**Figure 2:**
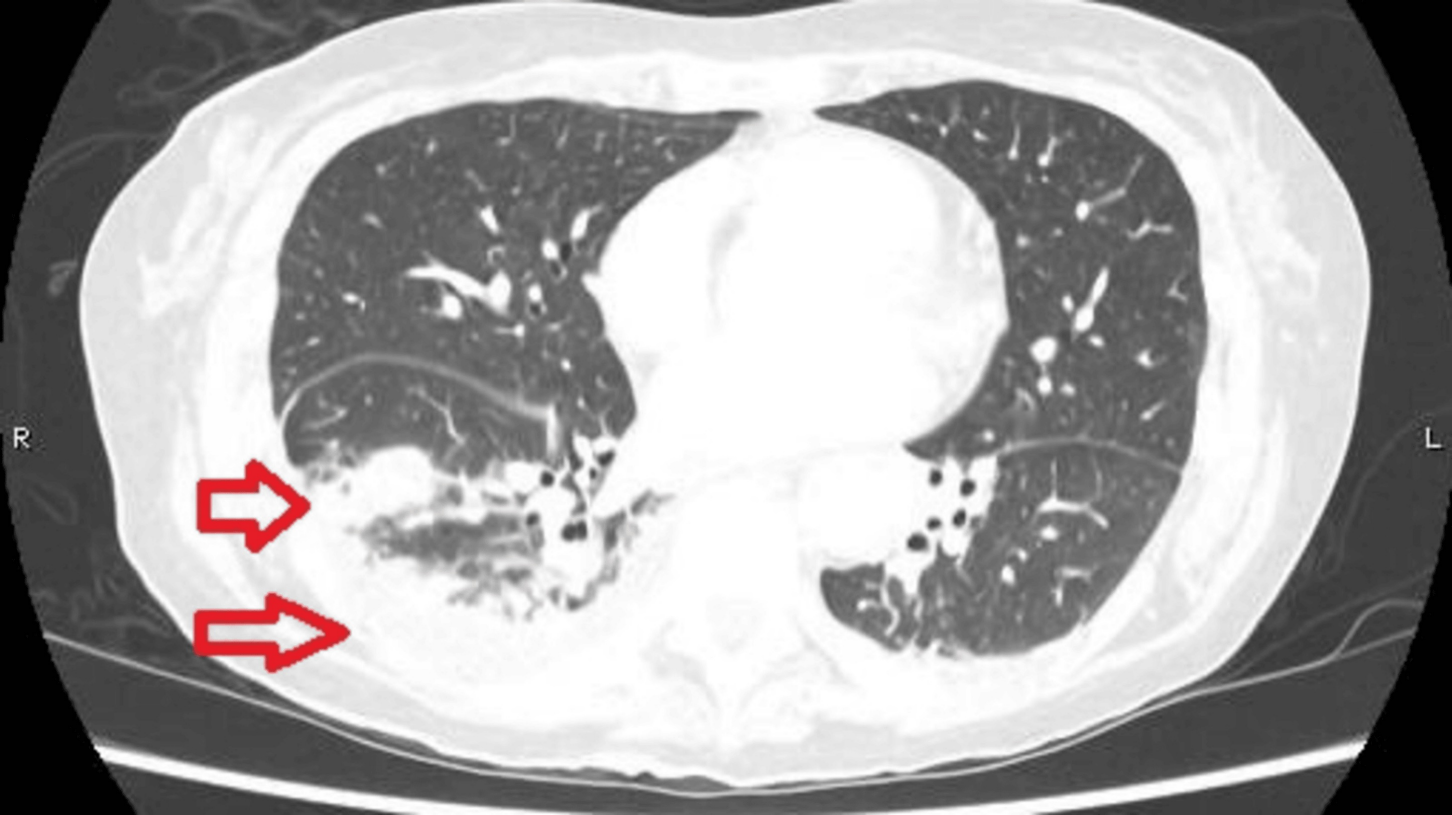
CTPA taken on admission showing multifocal pulmonary nodules along with bi-basal pleural thickening and small pleural effusion (red arrows). CTPA: computed tomography pulmonary angiogram

**Figure 3:**
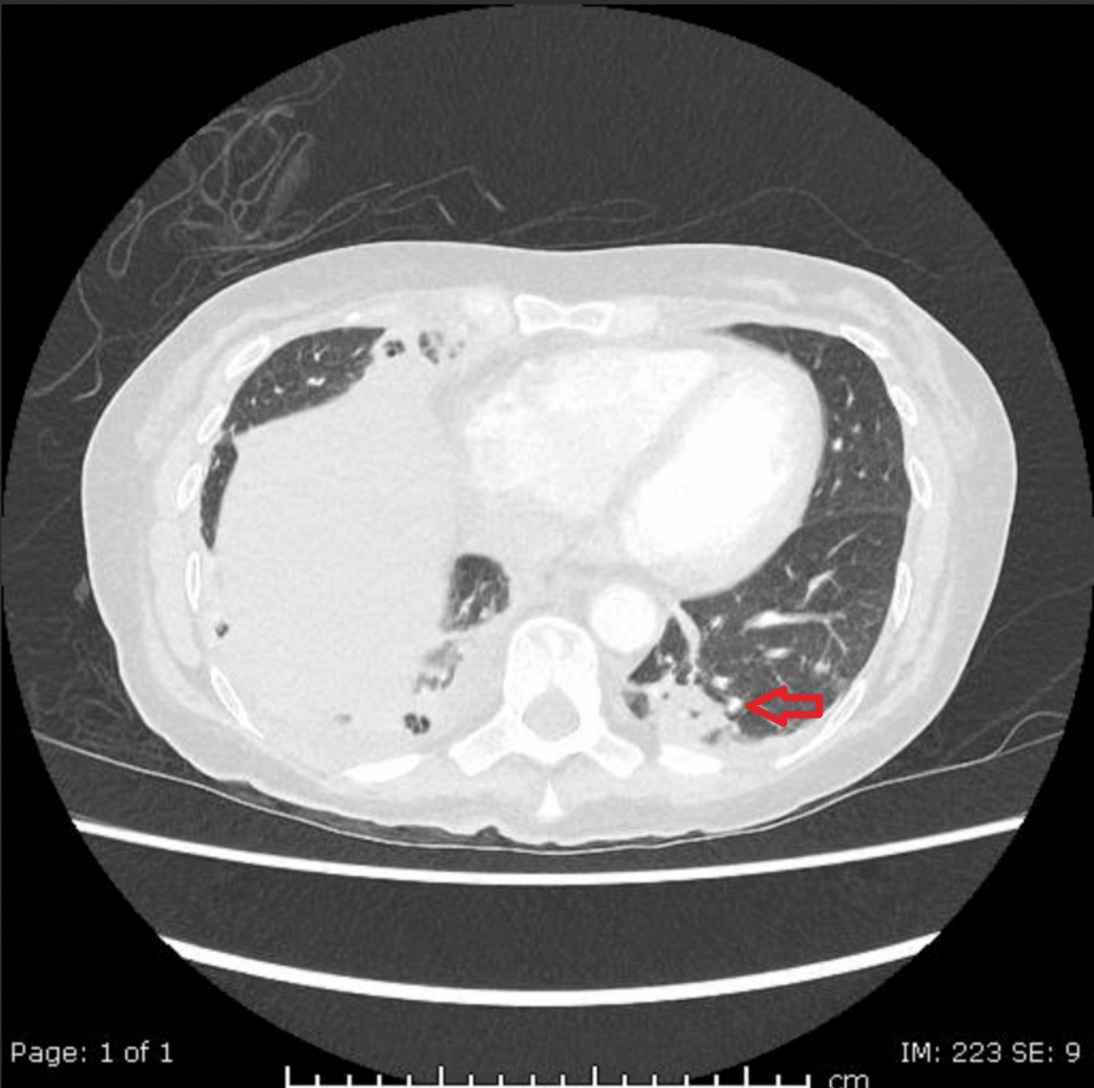
CTPA showing cavitating pulmonary nodule in the left lower lobe (red arrow). CTPA: computed tomography pulmonary angiogram

In view of her respiratory symptoms, a chest X-ray (Figure 1) and computed tomography pulmonary angiography (CTPA) (Figures 2, 3) were requested upon admission. These images reported bilateral basal infective/inflammatory changes more severe in the right lung than the left lung, bilateral pleural effusions with pleural thickening, and multifocal pulmonary nodules with few of them showing cavitating changes, and the differential possibility was infective, inflammatory, neoplastic or septic emboli.

**Figure 4:**
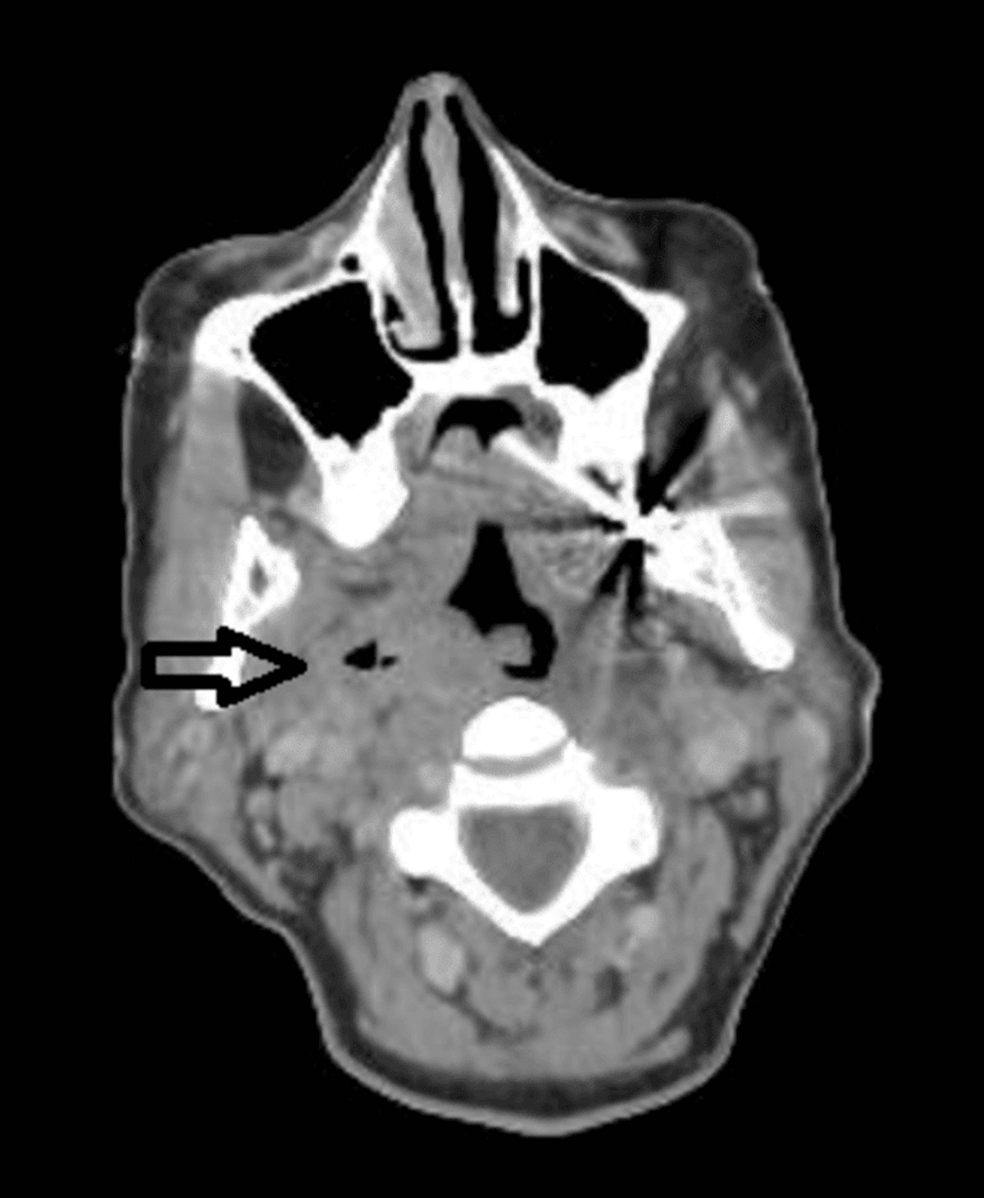
CT Neck showing thickening of right lateral wall of oropharynx, small peritonsillar collection containing gas, and soft tissue swelling causing mass effect (black arrow).

**Figure 5:**
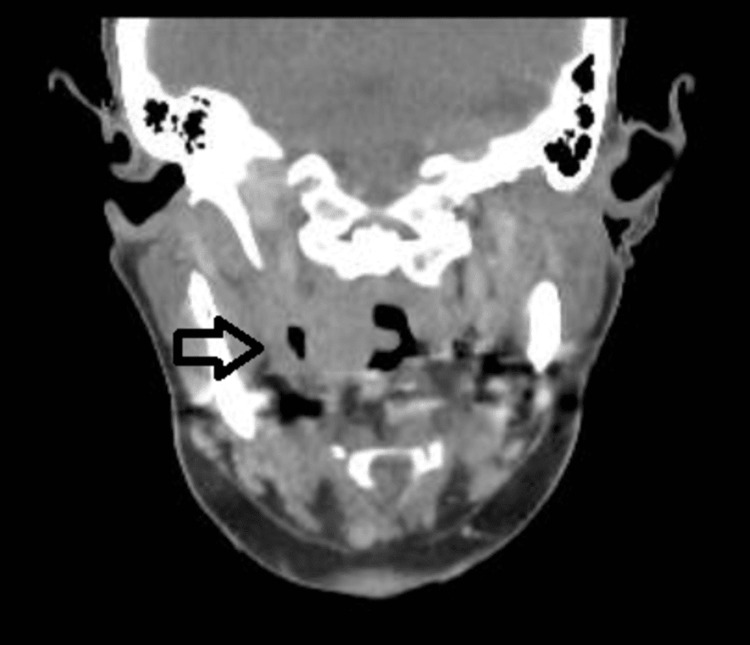
CT Neck showing peritonsilar collection containing gas (black arrow).

Following one day of incubation, blood culture grew gram-negative bacilli (GNB) from an anaerobic bottle. Although BIOFIRE® panels (BioFire Diagnostics, Salt Lake City, Utah, United States) did not show any GNB targets, on the third day, matrix-assisted laser desorption/ionization (MALDI) confirmed that the gram-negative bacteria were *F. necrophorum*. This marked a pivotal moment prompting the suspicion of Lemierre’s syndrome. As per minimum inhibitory concentration (MIC), the organism was susceptible to penicillin, co-amoxicillin, piperacillin-tazobactam, clindamycin, meropenem and metronidazole. Hence, piperacillin-tazobactam was continued. Despite adequate treatment with piperacillin-tazobactam, the patient continued to experience temperatures between 39-40.5 degrees Celsius. Consequently, on day 3, metronidazole 400 mg (per oral, twice a day) was added following the advice of the Microbiology team and further investigations were performed to rule out an abscess of unknown origin and the possibility of an underlying malignancy. A computed tomography (CT) scan of the neck (Figures 4, 5), requested by the Respiratory team under suspicion of Lemierre's syndrome to rule out internal jugular vein (IJV) thrombosis, did not show IJV thrombosis. However, it revealed thickening of the right lateral wall of the oropharynx and a suspected peritonsillar collection approximately 1.6 cm in size containing gas.

On day 4, a pleural tap was conducted, and samples were sent for biochemistry, culture, and cytology. Biochemical analysis revealed an exudative pleural effusion with normal pH and glucose levels. The culture results indicated the growth of skin commensals, which is highly suggestive of contamination. Additional imaging modalities including trans-oesophageal echocardiography, magnetic resonance imaging (MRI) of the spine and CT of the abdomen and pelvis were unremarkable.

**Figure 6:**
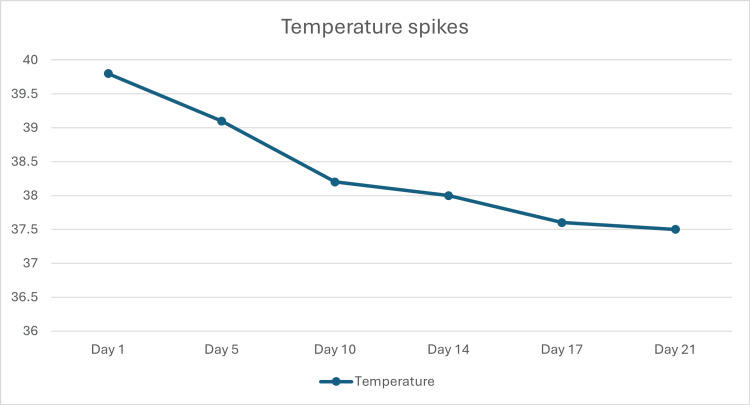
Trend of temperature spikes

By day 6, in response to persistent febrile episodes, the therapeutic protocol was modified by the Microbiology team, substituting piperacillin-tazobactam with meropenem (1 gm; three times a day) concomitantly administered with metronidazole. Regardless of this, she continued to have temperature spikes and this event further raised the suspicion of an underlying malignancy. At this stage, the laboratory verified that the pleural tap sample contained red blood cells and inflammatory cells and there were no malignant cells were seen. On day 10, following the Microbiology consultation, the antibiotic regimen was streamlined to piperacillin-tazobactam monotherapy, in response to the settling of fever. Figure 6 depicts the trend of temperature spikes during the course of admission.

On day 11, the Otolaryngology team performed a flexible nasendoscopy in an attempt to aspirate the peritonsillar collection. However, aspiration was not feasible as the collection had resolved, likely due to the antibiotic treatment administered to the patient in the preceding days. Despite the absence of IJV thrombosis, the diagnosis of Lemierre’s syndrome was then established based on clinical, microbiological and non-specific radiological findings.

Following one more week of piperacillin-tazobactam, the patient improved drastically and remained afebrile. Hence, after 21 days of IV antibiotics, she was then stepped down to an eight-week regimen of oral antibiotics comprising amoxicillin 1 gm three times a day and metronidazole 400 mg three times a day. Additionally, she also received a thrombopoietin receptor agonist to stimulate platelet production to address a low platelet count under the guidance of the Haematology team. This was suspected to be Idiopathic thrombocytopenia secondary to infection as the coagulation screen did not meet the International Society of Thrombosis and Haemostasis criteria.

Alongside medical intervention, the Respiratory Physiotherapy team played a crucial role in the patient’s recovery providing supportive measures such as cough packs and diaphragmatic breathing exercises, which had a positive impact on the patient’s symptoms.

**Figure 7:**
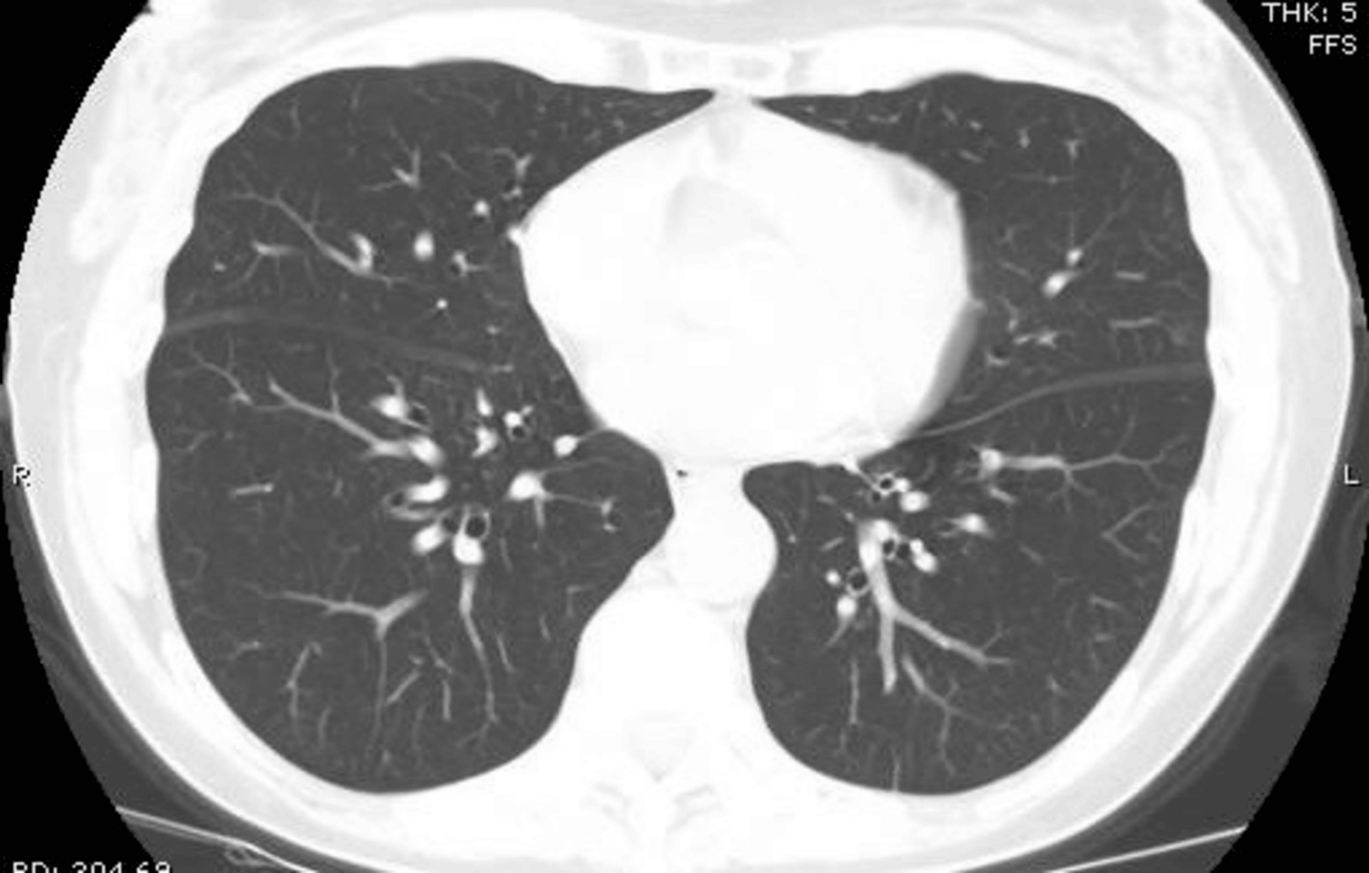
Follow-up CT Thorax taken three months after discharge showing complete clearing of ground glass nodules.

Throughout the rest of her hospital stay and subsequent outpatient follow-up, the patient demonstrated significant clinical improvement with resolution of pulmonary consolidations and effusions noted on serial imaging studies. Symptomatically, the patient reported feeling well and resumed normal activities. Repeat imaging, including CT of the neck and thorax at six weeks and CT of the thorax at three months post discharge (Figure 7), demonstrated marked improvement with minor residual changes, indicative of successful treatment response. The patient remained asymptomatic, with no recurrence of symptoms during follow-up visits at six weeks and three months.

## Discussion

Lemierre’s syndrome remains a diagnostic challenge due to its varied clinical presentation [1,3]. Although there can be a constellation of findings, a systematic review of 84 studies published between 1950 and 2007 found the most common first symptoms at presentation were sore throat (33%), cervical lymphadenopathy (23%), and neck pain (20%)[2]. The following criteria are considered strong evidence to confirm the presence of Lemierre’s syndrome: deep neck infections, subsequent septicaemia, thrombophlebitis of the IJV, and metastatic infections (ascending or descending septic emboli) [1,3-6]. Lemierre's syndrome is primarily caused by *F. necrophorum* and other pathogens such as various *Fusobacterium* species, *Enterobacteriaceae*, and *Streptococcus* species, including rare cases of *Staphylococcus aureus* [6-17].

Understanding the diverse microbial aetiology is crucial for accurate diagnosis and appropriate management of Lemierre's syndrome. In the majority of Lemierre's syndrome cases, the primary infection originates from pharyngitis involving the palatine tonsils or peritonsillar tissue [13]. Less frequently, primary sources of infection may include odontogenic infections, mastoiditis, otitis media, sinusitis, and parotitis [10]. Following primary infection, there is subsequent local invasion into the pharyngeal space and IJV, leading to septic thrombophlebitis typically occurring within one to three weeks [6].

Possible mechanisms of Lemierre’s syndrome include hematogenous spread through the tonsillar vein, invasion of the peritonsillar region, or dissemination to the adjacent lateral pharyngeal space through lymphatics [6]. Alternatively, alterations in the pharyngeal mucosa during primary bacterial or viral infections may facilitate local invasion by *F. necrophorum*, allowing direct extension through the fascial planes of the neck to the IJV, causing various complications and ultimately death due to septic shock [10]. The resulting bacteraemia can cause neurological complications in 30% of cases, including meningitis, venous sinus thrombosis, abscesses and stroke. The lungs are most affected in 85% of cases [8].

Early diagnosis of Lemierre’s syndrome is instrumental in initiating prompt and targeted therapy, thereby mitigating the risk of complications. The delay in diagnosis can result in the progression of septicaemia, septic embolization, and multi-organ dysfunction with mortality of 2-18% [2]. Although sepsis is a hypercoagulable phase and prompt use of anticoagulation is warranted, anticoagulation in Lemierre's syndrome does not affect thrombosis outcomes as per retrospective studies. Aggressive and prompt antimicrobial therapy remains the standard of care for this life-threatening disorder [18].

Empiric therapy for Lemierre's syndrome should target *F. necrophorum* and oral streptococci, using antibiotics resistant to beta-lactamase [19], such as piperacillin-tazobactam, carbapenems, or ceftriaxone plus metronidazole. Therapy should be tailored based on culture results, with the duration generally being at least four weeks, including two weeks of intravenous treatment, and adjusted for complications. The clinical response to treatment may be slow, even with appropriate antimicrobial therapy; however, if persistent bacteraemia or worsening occurs, repeat imaging, susceptibility testing, and potentially additional interventions may be necessary [20].

One of the key aspects of this case is the nonspecific and diverse symptomatology exhibited by the patient, including sore throat, chest pain, haemoptysis, gastrointestinal symptoms and back pain. These symptoms, individually, may not immediately raise suspicion for Lemierre’s syndrome. The timely utilization of basic diagnostic tools such as blood cultures and imaging studies played a crucial role in confirming the diagnosis. Imaging studies including chest X-ray and CT helped identify pulmonary complications, aiding in the comprehensive assessment of the disease burden. Nevertheless, it was the astuteness of the healthcare team that initiated further investigation, ultimately leading to the diagnosis of Lemierre's syndrome. This highlights the importance of thorough clinical evaluation and vigilance in recognizing subtle clinical clues that may point towards Lemierre’s syndrome.

## Conclusions

This case underscores the importance of early recognition and diagnosis of Lemierre’s syndrome, especially in patients with uncommon presentations like elderly females with varied symptomatology unresponsive to intensive antibiotic therapy and atypical findings like pulmonary nodules with cavitating changes suspicious of neoplasm in the absence of IJV thrombosis. Heightened clinical suspicion, coupled with timely utilization of diagnostic modalities to detect both IJV thrombosis and disseminated septic emboli, is essential in preventing morbidity and mortality associated with this potentially life-threatening condition. Continued education and awareness among healthcare providers are paramount to ensure prompt diagnosis and optimal outcomes in patients presenting with Lemierre’s syndrome.
